# Adrenocortical carcinoma with skeletal metastases in a postmenopausal woman

**DOI:** 10.4103/0971-5851.56332

**Published:** 2009

**Authors:** Shila Mitra, Suparna Ghosh Roy, Prabir Kumar Sur

**Affiliations:** *Department of Radiotherapy, Medical College Hospital, 88, College Street, Kolkata - 700 073, India*

**Keywords:** *Adrenocortical carcinoma*, *androgen secreting tumors*, *mitotane*

## Abstract

Adrenocortical cancer is a very rare tumor with a poor prognosis. About half of them are hormone-secreting tumors. In most cases, hormonal investigations reveal an excess secretion of steroids, mostly cortisol and androgens. A 54-year-old lady presented with history of pain in left shoulder and leg for 6 months and features of virilization. CT-guided fine-needle aspiration cytology of an abdominal mass revealed the presence of a carcinoma of the left adrenal cortex. A whole-body radionuclide bone scan revealed increased uptake in the left clavicle and left femur. The patient has received palliative radiotherapy for the skeletal lesions and 3 cycles of palliative chemotherapy at present.

## INTRODUCTION

Carcinoma arising from the adrenal cortex is a very rare malignancy with an extremely poor prognosis.[1] It has an incidence of about 1-2 per million population[2] and accounts for 0.02% of all malignancies. It is bilateral in 10% of cases, with a bimodal age distribution.[3] The first usually occurs between 2 and 5 years of age; and the second, between 40 and 50 years. Adrenocortical cancers (ACCs) represent about 0.002% of childhood malignancies. There is no racial predilection in the occurrence of such tumors, though the incidence of pediatric ACCs in Brazil is said to be 3 times that in the rest of the world (3.4-4.2 cases per million children below the age of 15 years).[2] Though there is no sexual predilection of occurrence of ACCs, females have a slightly increased incidence of functioning tumors. It has been observed that at the time of presentation, 30% to 35% of patients present with metastatic disease.[4] Here we present the case of one such patient who presented to us with features of virilization and bone pain.

## CASE REPORT

A 52-year-old female patient presented to us with pain in the left thigh and inability to walk for the past 3 months. She had also noticed a painless hard lump over the left clavicular region for the last 6 months. On further inquiry, it was revealed that the lady had gradually developed temporal alopecia, appearance of fine facial hair, as well as hair on the arms and upper back. There was also a deepening and hoarseness of her voice for the last 3 years. She gave no history of any flushing attacks, diarrhea, sweating or palpitation. The patient has 6 children and had attained menopause 6 years back. There was no significant past medical or surgical history or history of intake of any medication.

**Figure 1:**
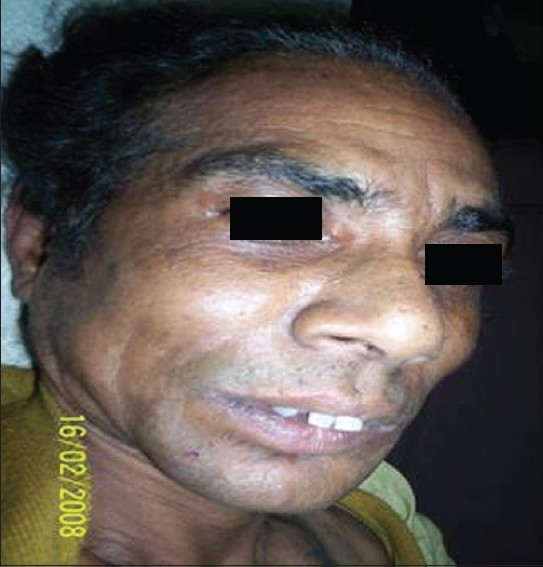
The patient with masculine facial features

On examination the patient had features of hirsutism, suggested by facial appearance resembling that of a male with facial hair, with hair also present on her upper limbs and back [Figure 1]. She also had temporal baldness, coarse hands resembling male hands, atrophy of the breasts and clitoromegaly. There was also pitting nontender edema of the left lower limb after an incident of trivial trauma. Examination per abdomen revealed a nontender mass in the left lumbar area and nontender hepatomegaly. Her blood pressure measured on several occasions was not raised.

Results of hematological tests were within normal limits. Liver enzymes were not raised (except a mildly elevated serum alkaline phosphatase-208 U/L). Serum urea and creatinine were within normal limits. Fasting blood sugar was 116 mg%. Serum Na^+^ (139 mmol/L), K^+^ (4.1 mmole/L) were not raised either, but serum 17 dehydroepiandrostenedione (17 DHEA) was >10 ng/ mL (normal values, < 0.9-3.6 ng/ mL). Twenty-four-hour urinary cortisol was marginally raised, 227.87 μg per 24 hours (normal value, 28.50-213.70 μg per 24 hours).

**Figure 2:**
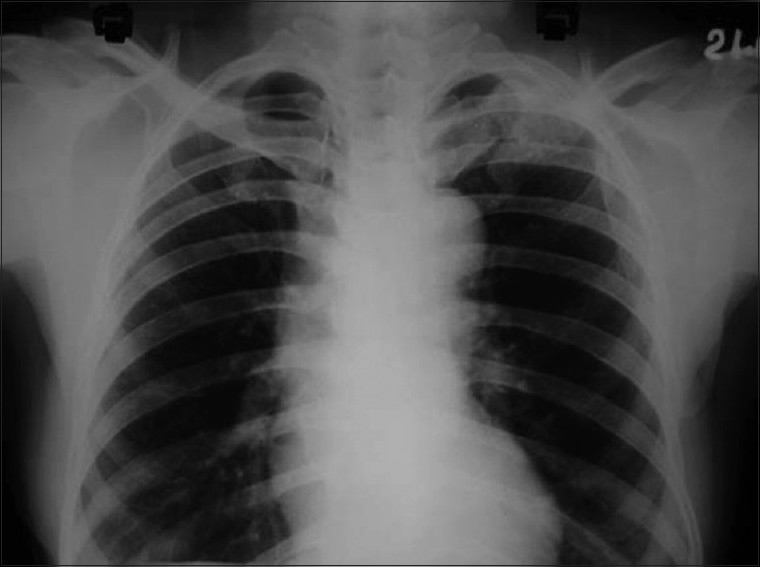
PA view of chest showing fracture of left clavicle

**Figure 3:**
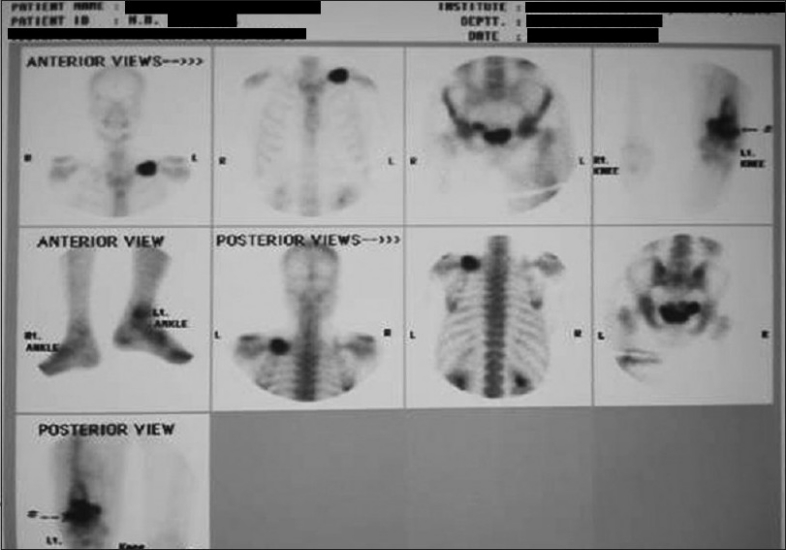
Whole-body bone scan using Tc 99 m showing increased uptake by left clavicle and distal third of left femur

**Figure 4:**
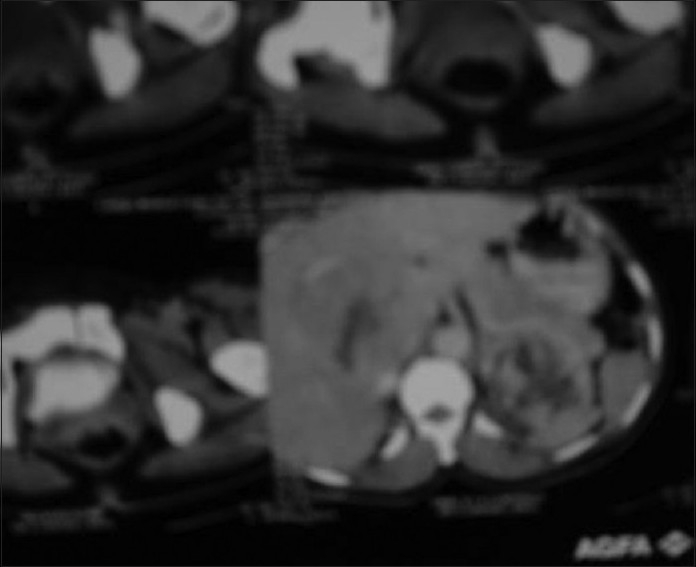
Computerized tomography scan of abdomen showing a heterogeneous suprarenal mass on the left side and a heterogeneous space occupying lesion in the liver

Skiagram of postero-anterior view of the chest revealed a fracture of the left clavicle [Figure 2]. Computerized tomography (CT) scan of the thorax was also advised, but the patient was unable to afford it. X-ray of the left limb showed a circumscribed lytic lesion in the lower shaft of the left femur. A whole-body bone scan (isotopic skeletal survey) with 99 m technetium-methylene diphosphonate revealed abnormally increased uptake over the left clavicle and distal end of left femur [Figure 3]. CT scan of the whole abdomen revealed multiple ill-defined hypodense enhancing space occupying lesions of varying sizes involving both lobes of the liver [Figure 4]. There was a 7.9 × 6.8 cm irregular heterogenous enhancing lesion of the left adrenal gland displacing the upper pole of the left kidney. The adrenal gland of the right side showed no abnormality. There was no retroperitoneal lymphadenopathy. ECG was within normal limits.

**Figure 5:**
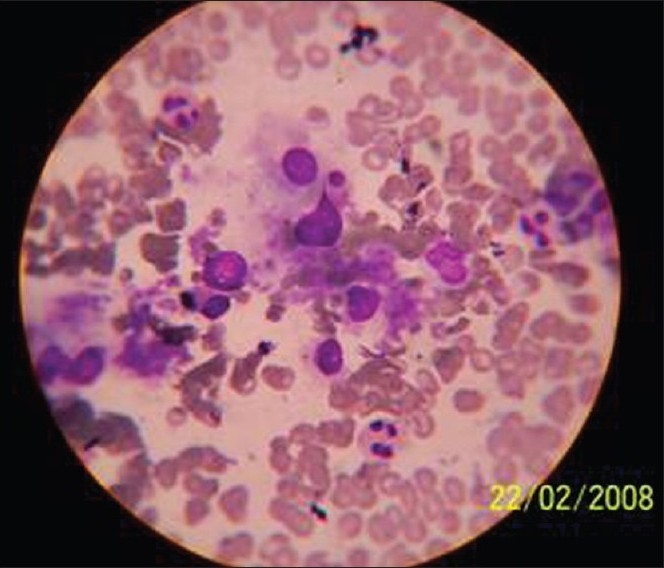
Fine-needle aspiration cytology of the left adrenal mass showing carcinomatous cells

Ultrasonography (USG)-guided fine-needle aspiration cytology (FNAC) from the left adrenal swelling showed malignant cells, possibly adrenocortical carcinoma [Figure 5]. FNAC from the left clavicular swelling revealed metastatic malignancy.

The patient was treated by giving external radiation to the areas with skeletal metastases. The left clavicular lesion was treated by a direct AP field to a dose of 2000 cGy in 5 fractions. The left femoral lesion was treated using the same dose. External radiation was given by Co 60 (Theratron 780C). Due to the presence of multiple hepatic metastases involving both lobes of the liver and after consultation with the concerned surgeon, it was decided that operative intervention would not be feasible. After completion of radiotherapy, the patient received 3 cycles of chemotherapy with Inj. cisplatin @ 80 mg/m^2^ Day 1 and Inj. doxorubicin @ 40 mg/m^2^ Day 1 every 21 days. A repeat CT scan of the abdomen at the end of 3 cycles of chemotherapy showed no response of the tumor to this regime.

## DISCUSSION

Carcinoma of the adrenal cortex is a very rare malignancy and constitutes about 0.05% to 0.2% of all malignancies and has a prevalence of 4-12 per million of adult population. These carcinomas account for 0.2% of all cancer deaths. Adrenal cortex tumors may be functioning or nonfunctioning. About 50% of such tumors are functioning ones, with most presenting with Cushing′s syndrome.[5] Men tend to develop nonfunctioning carcinomas more commonly than women. ACCs have a bimodal age distribution, the first with a peak at age below 5 years and the second with a peak between the fourth and fifth decades of life. They are often associated with carcinoma breast, cancer lung and sarcomas forming a part of a complex syndrome. Genetic changes are more common in malignant than benign adenomas. Mutations in *p53* gene are common and may be used as an optional diagnostic test. About 10% of malignant tumors produce features of masculinization due to testosterone production, whereas the rest secrete cortisol, aldosterone or nothing. Excessive cortisol secretion gives rise to features of Cushing′s syndrome, whereas excess mineralocorticoids lead to mild increase in blood pressure. Co-secretion of cortisol and androgen is the most common presentation and is highly suggestive of malignancy.[6] In our case, serum androgen was much higher than the normal range, cortisol secretion was only slightly raised and typical features of hypercortisolism were also absent. Solely androgen secreting tumors are rare, and even 50% of such tumors are benign.[7]

Clinically, virilization suggests malignancy.[3] Patients most commonly present with features of rapidly developing Cushing′s syndrome with or without virilization. Estimation of plasma cortisol or 24-hour urinary cortisol after overnight dexamethasone suppression test establishes hypercortisolism. Both adrenocortical cancers and ectopic secretion of ACTH cause no suppression of cortisol secretion after both low dose and high dose of overnight dexamethasone. Histopathological diagnosis is obviously superior to cytological studies. The Weiss criteria (a histological algorithm) should be used in histological studies of ACC tumors.[2] A Weiss score > 2 is indicative of ACC.[5,6] Of the 9 criteria in the Weiss criteria, a mitotic rate >5 per 50 high-power field, atypical mitotic figures and venous invasion are the 3 most often found criteria in ACCs.[5,6] But due to lack of histologic examination, we were unable to assess the mitotic index or the Weiss criteria in this rare tumor.

Imaging studies such as CT scan/MRI also help to detect the nature of adrenocortical tumors to some extent by not only stating the size but also by the appearance on CT scan.[2] Measuring the Hounsfield units on unenhanced CT scan can give an approximation regarding the nature of the mass, with a value of 10 or less as being characteristic of benign lesions. However, recent studies indicate that using delayed contrast-enhanced CT scan is of help in assessing the nature of the lesion by analyzing the wash-out time of the contrast. An enhancement washout of of less than 50% and a delayed attenuation of more than 35 HU should arouse suspicion of malignancy.[2] Gadolinium-enhanced magnetic resonance imaging (MRI) is equally effective in distinguishing benign from malignant lesions.[5] The fat content of the tumor helps to distinguish between malignant and benign lesions. Fluorodeoxyglucose positron emission test (FDG PET) studies are also promising in staging the disease and detecting distant metastases. The 11 C metomidate PET is especially helpful in distinguishing adrenal lesions from other masses.

ACCs are classified by the Macfarlane classification or Sullivan modification of Macfarlane classification and the TMN classification is an adaptation of the aforesaid staging system. Among the various prognostic factors, tumor staging is one of the important clinical parameters which affect prognosis of ACCs.[8] Also the number of tumoral organs at the time of first metastasis and mitotic index can predict different outcomes of the tumor.[8] Patients having less than two tumoral organs with metastases and less than 20 mitoses per high-power field in the primary tumor can be predicted to have a relatively better 5-year survival compared to patients having metastatic diseases with increased tumor burden.[8] In our patient′s case, there were multiple skeletal metastases and also hepatic metastases. Diagnosis in this case was established by clinical features, cytology and biochemical parameters. Malignant ACCs usually weigh more than 100 g and have diameters >6 cm in the greatest dimension. The adrenal tumor in the discussed case measured more than 6 cm in the greatest dimension on CT scan.

The mainstay of treatment of not only primary ACCs in stage I to III disease but also in selected cases of locally recurrent, residual disease and in limited metastatic lesions is surgical resection.[3] R0 resection is associated with superior prognosis. Extensive en bloc resection with lymphadenectomy may be required in certain cases. Surgical resection may be considered in metastatic disease if both the primary and metastatic lesions are to be removed completely. With the advent of minimally invasive surgery, laparoscopic adrenalectomy is performed for tumors <5-6 cm without any invasion to surrounding structures.[4] Surgical fixation may also be performed in patients with metastases in weight-bearing bones. But in our case, surgery could not be done as the patient had presented with multiple metastases involving both lobes of the liver as well as the left clavicle and left femur. Hence we were also unable to assess the weight of the tumor.

There is evidence that radiotherapy may be used as palliative treatment in bone, brain metastases, as well as in postoperative recurrences.[1] Radiotherapy to the tumor bed after surgical resection in early stages is controversial. Radiofrequency ablation is another option for patients that are poor candidates for surgery but have metastatic lesions <5 cm.[9]

We had advised our patient to take mitotane along with palliative chemotherapy. As she was unable to afford mitotane, she received 3 cycles of chemotherapy with doxorubicin and cisplatin, but to no effect. Though mitotane (a synthetic derivative of the insecticide DDT) has been used in trials as an adrenolytic agent, its toxicities have precluded its usage in clinical practice. Mitotane has been recommended in patients that have undergone complete surgical resection, as the rate of recurrence is quite high in ACCs (50%-60%)[9] and use of this agent has led to increased recurrence-free survival. [2,10] A threshold level of 14 g/dL of serum mitotane has been suggested to achieve any response, though lower levels have also been seen to obtain any response. A daily dose of 2 to 5 g is recommended to achieve a clinical response. Higher daily dose of mitotane mandates a biweekly monitoring of serum levels.[2] Gastrointestinal and central nervous system toxicities are the two most common adverse effects noted with the use of this drug. Steroid hormone supplementation should be used where mitotane is used as adjuvant therapy. Adrenostatic drugs may be used along with mitotane initially to block the steroidogenic enzymes.

Single-agent chemotherapeutic drugs used in adrenocortical carcinomas give a low response rate for a limited duration. Certain combinations[3,4] like cisplatin-doxorubicin, cisplatin-doxorubicin-etoposide, doxorubicin-mitotane, cisplatin-etoposide-mitotane, cisplatin-doxorubicin-mitotane are tried in adrenocortical carcinoma with response rates varying between 12% and 54%.[4] Higher response rates are found with mitotane combinations. The combination of cisplatin-doxorubicin-etoposide-mitotane has produced clinical response rates of about 50% even in advanced cases.[9] Combination of mitotane and streptozotocin has been showed to achieve good clinical response with limited toxicity.

Prognosis in advanced ACCs is very poor, with a 5-year survival of 10% to 25% and a mean survival of 5 to 18 months. At the end of 6 months from diagnosis, our patient is still alive with subjective improvement of her symptoms.

Present treatment modalities are often disappointing in the management of advanced cases of adrenocortical carcinomas, and it is hoped that further molecular studies of this rare tumor may offer new treatment options.
